# Synthesis and Characterization of Inulin-Based Responsive Polyurethanes for Breast Cancer Applications

**DOI:** 10.3390/polym12040865

**Published:** 2020-04-09

**Authors:** Gustavo A. Molina, Alberto Elizalde-Mata, Ángel R. Hernández-Martínez, Gerardo Fonseca, Martha Cruz Soto, Ángel Luis Rodríguez-Morales, Miriam Estevez

**Affiliations:** 1 Posgrado en Ciencia e Ingeniería de Materiales, Centro de Física Aplicada y Tecnología Avanzada (CFATA), Universidad Nacional Autónoma de México (UNAM), Blvd. Juriquilla 3000, Querétaro 76230, Mexico; gustavomolina21@gmail.com (G.A.M.); joshep_mata@outlook.com (A.E.-M.); 2 Centro de Física Aplicada y Tecnología Avanzada (CFATA), Universidad Nacional Autónoma de México (UNAM), Blvd. Juriquilla 3000, Querétaro 76230, Mexico; arhm@fata.unam.mx (Á.R.H.-M.); gerardo@fata.unam.mx (G.F.); alrodriguez@fata.unam.mx (Á.L.R.-M.); 3 Universidad del Valle de México, Campus Querétaro, Blvd. Juriquilla 3000, Querétaro 76230, Mexico; martha.cruzso@uvmnet.edu

**Keywords:** inulin, polysaccharide-based polyurethane, breast cancer, drug delivery, biomarker

## Abstract

In this study, new polyurethanes (PUs) were prepared by using inulin and polycaprolactone as polyols. Their structure and morphology were determined by Fourier transform infrared spectroscopy (FTIR), Raman dispersive spectroscopy, Nuclear magnetic resonance spectroscopy (^1^H NMR and ^13^C NMR), and scanning electron microscopy (SEM), whereas their mechanical properties were evaluated by a universal testing machine. Additionally, their water uptake, swelling behavior, and degradation were evaluated to be used as drug delivery carriers. Therefore, an anti-cancer drug was loaded to these PUs with 25% of loading efficiency and its release behavior was studied using different theoretical models to unveil its mechanism. Finally, the ability of the new PUs to be used as a clip marker in breast biopsy was evaluated. The results clearly demonstrate that these PUs are safe and can be used as intelligent drug release matrices for targeted drug delivery and exhibits positive results to be used for clip marker and in general for breast cancer applications.

## 1. Introduction

Breast cancer is the leading cause of cancer-related deaths among women and is one of the most common tumors, which represents 31% of total cases affecting the female population [1,2]. The increase in breast cancer incidence leads to the need of improving diagnostic and therapeutic tools [3]. In some cases, it is necessary using clip markers—small objects made of metal (e.g., titanium) or ceramic that are inserted into the tumor before starting the therapy of patients—to identify the specific part of the body where a biopsy was performed. Those markers have high visibility in imaging for further studies such as ultrasound or magnetic resonance imaging (MRI) scan. However, a second surgery is required to remove the marker clip, with the inherent risks and possible complications for the patients.

On the other hand, recent developments in polymer science have provided great opportunities for manufacturing-controlled drug delivery systems from polyurethanes (PUs). Biodegradable PUs are extensively used as suitable candidates for intelligent drug delivery, such as pH-responsive PUs as smart drug delivery materials [4,5,6,7,8].

Polyurethanes (PUs) are an important class of polymers synthesized through a polyaddition reaction between polyols and polyisocyanate that forms urethane linkages. They are formed by a urethane linkage (–N–CO–O) in the main chain as a characteristic feature and this linkage is analogous to the peptide bonds in the structure of proteins [9]. In comparison with other conventional materials, polyurethanes possess remarkable properties such as moisture permeability, thermal conductivity, low density, high strength to weight ratio [9,10,11,12]. PUs are used in biomedical science for new applications due to their specific features. For example, they have been used for replacement of biological materials in human body such as, valves, temporary scaffolds, and breast implants [13]. As mentioned before, polyurethanes also have been extensively studied for biomedical applications as hydrogels and electrospun fibers for controlled release of drugs and biological molecules due to their good mechanical strength, good stability flexibility, and biocompatibility [14]. Those properties are directly related to the type and nature of precursors, chain extenders, and synthesis route.

Other research works offer alternative eco-friendly pathways, by modifying or substituting precursors. For example, PU derived from lysine has been reported as biocompatible and acceptable. Polysaccharides are source of hydroxyl groups, that alternative offers mimetic properties and controllable degradable rate without losing mechanical properties. Polysaccharides have a wide range of structural diversity (linear to highly branched structures) attributed to different functional moieties. Hydrophobicity, rigidity, biocompatibility, and bioactivity of polysaccharides are essential features for biomaterials [15,16]. 

In this study, we propose synthesize polyurethanes using inulin as a precursor. Inulin is a water-soluble storage polysaccharide that belongs to a group of non-digestible carbohydrates called fructans. Inulin has attained the Generally Recognized as Safe (GRAS) status in USA and it is extensively available in about 36,000 species of plants, among which, chicory roots are considered as the richest source of inulin [17]. Commonly, inulin is used as a prebiotic, fat replacer, sugar replacer, texture modifier, and to develop functional foods in order to improve health (due to its beneficial role in gastric health) [18,19].

## 2. Materials and Methods 

### 2.1. Chemicals

Polycaprolactone diol (PCL-diol, M*_n_* ~ 2000), tin(II) 2-ethylhexanoate (Sn-Oct), hexamethylene diisocyanate (HDI: >98%), 1,4-diaminobutane (putrescine: 99%), 2-propanol (IsOH, anhydrous: 99.5%), inulin from dahlia tubers (n ≈ 36, M*_r_* ~ 5000), ferric chloride hexahydrate (FeCl_3_·6H_2_O, 97%), ferrous chloride (FeCl_2_, 98%), and doxorubicin hydrochloride (DOXO, for HPLC: 98–102%) were purchased from Sigma Aldrich Co. Mexico; ammonium hydroxide (NH_4_OH, ~29%), sodium citrate tribasic dihydrate (Na_3_C_6_H_5_O_7_·2H_2_O, ~98%), 2-propanol were purchased from J.T. Baker. Ethylene dichloride (ACS reagent grade) was purchased from Macron Chemicals and Hartmann physiological solution was purchased from the local drugstore. All reactants were used as received.

### 2.2. Synthesis of Inulin-Based Polyurethane (PU–INU)

The novel inulin-based polyurethane was synthetized using a conventional two-step method in which HDI was used as the isocyanate precursor, PCL-diol and inulin (INU) as the poly-nucleophile (active hydrogen moieties), Sn-Oct as catalyst and putrescine as chain extender. All synthesis were performed using ethylene dichloride as solvent and according to the data shown in Table 1.

First, polyol precursor was dissolved in 40 mL of ethylene dichloride, previously poured on a three-necked flask with a magnetic stir bar and thermometer (provided with a water cooling condenser in order to prevent evaporation of the solvent) at 40 °C and subjected to vigorous stirring to secure dissolution. Then, the described setup was heated up to 75 °C in order to add the catalyst (Sn-Oct) through injection and the mixture was kept in these conditions for 20 more minutes. After the elapsed time, the first polymerization step (pre-polymer formation) was initiated by adding the isocyanate (HDI) through injection using a syringe, to maintain stirring and temperature conditions (75 ± 2 °C) of the setup and left to react for 3.5 h.

The second polymerization step was performed by maintaining vigorous stirring, reducing the temperature of the setup to 60 °C and adding in the setup an amine as a chain extender (putrescine—175 μL) through injection using a syringe and left to react for additional 30 min under the described conditions to form a poly(urethane-urea) polymer. Finally, the reaction was cooled to room temperature and the formed polymer was precipitated using 2-propanol for 24 h. Then the solvent was decanted and swollen polymer was placed in several falcon tubes in which clean 2-propanol was added, the mixture was placed on a centrifuge (Centrificient IV, CRM-Globe, TX, USA) and a centrifugation cycle of fifteen minutes using 3000 rpm (to precipitate the polymer), then the 2-propanol was decanted, this procedure was repeated two more times and were initiated by adding new solvent after decantation. 2-propanol was selected as the solvent to be used in order to eliminate free isocyanate groups (considered as toxic) [20,21].

Finally, samples were labeled as shown in Table 1 and each of the swollen polymers were placed on a personalized silicone mold to cast films for their characterizations. The polymer cast were dried at 40 °C using a vacuum drying oven ADP 200C (Yamato Scientific Company LTD., Tokyo, Japan) under controlled atmosphere.

### 2.3. Characterization of PU–INU

#### 2.3.1. Structural Characterization

The structural characterization of the polymers was performed through three different techniques. Fourier Transform Infrared spectroscopy (FT-IR) and Raman dispersive spectroscopy were used to observe if there are residues of isocyanate groups and the characteristic bands for polyurethanes. Nuclear magnetic resonance spectroscopy (^1^H NMR and ^13^C NMR) was also used to determine the molecular arrangement of inulin on the new polyurethane-based material.

The FT-IR characterization of polyurethanes was performed by a Vector 33 Spectrometer (Bruker Biospin Corporation, Billerica, MA, USA) under attenuated total reflectance (ATR) mode from 400 to 4000 cm^−1^ and RAMAN spectroscopy was performed using a Senterra apparatus (Bruker Biospin Corporation, Billerica, MA, USA) equipped with λ = 685 nm laser and FT-Raman (Nicolet 910) with λ = 1064 nm in the laser, coupled with an Olympus microscope. For both spectroscopies, no further preparation of samples is required.

For ^1^H NMR and ^13^C NMR, the analysis was carried out on a Bruker Avance III HD 500 MHz instrument (Bruker Biospin Corporation, Billerica, MA, USA), using deuterated dimethyl sulfoxide (DMSO-6d) as solvent for the PU–INU and tetramethylsilane as internal standard at 25 °C. The chemical shift data is reported as part per million (ppm) *δ* scale.

#### 2.3.2. Thermogravimetric Analysis

Thermogravimetric analysis (TGA) was done to determine thermal degradation of polyurethanes and in order to observe changes in chemical composition of PU–INU. With this purpose in mind, a TGA/DSC Model 2 StaRe System (Mettler-Toledo Intl. Inc., Columbus, OH, USA) was used. Samples of 3–6 mg were used and heated from 20 to 900 °C with a scan rate of 10 °C/min under a controlled nitrogen atmosphere and a flow rate of 40 mL/min at normal conditions of temperature and pressure.

#### 2.3.3. Physical Properties Characterization

Three different techniques were used to evaluate physical properties of the PU–INU based materials: mechanical characterization, swelling and analysis of hydrolytic degradation.

For mechanical properties evaluation, a tensile test on PU–INU films was performed using a Zwick/Roell Z005 equipment (Ulm, Germany) according to the standard ASTM D638-03, using a 500 N, and data was obtained employing TestXpert Intelligent Testing 12.0 software. Each test was carried out at 25.2 °C, 46% of relative humidity using a speed of 10 mm·min^−1^ until fracture was produced.

Swelling ratio measurements of the polyurethanes were performed using a gravimetric analysis and swelling ratio (*S_r_*, %) was calculated using Equation (1):

*S_r_* (%) = [(*w_s_ − w*_0_)/*w*_0_] × 100 (1)

where *w*_0_ is the initial polyurethane mass when it is dry and *w_s_* is the mass of the polyurethane when it is swollen. A known pre-weighted and standardized 0.5 cm^2^ film fractions were soaked in deionized water at room temperature and measured at different elapsed time intervals until the equilibrium swelling ratio (*ES_r_*) was reached.

Effect of temperature and *pH* on swelling properties were evaluated for each PU–INU films with the following conditions: (i) for temperature-dependent swelling, the dried films were immersed into deionized water with an initial temperature of 5 °C and gradual increments of 5 °C were performed until 40 °C was reached and (ii) for pH-dependent swelling, the dried films were exposed in separate pH buffer solutions (pH values of 4.0, 7.0, and 10.0) at room temperature. In both cases, the swelling ratio was measured gravimetrically until the polyurethane reached *ES_r_* and calculated using Equation (1).

Degradation assessment was performed as reported elsewhere [22] in a static immersion of the samples simulating two physiological conditions such as pH by using a Hartmann solution (pH = 7.4) and temperature at 37 °C using a custom incubator controlled by a PDI-TC4S Temperature Controller (Autonics Corporation LTD., Busan, Korea). For the evaluation, a pre-weighed 0.5 cm^2^ standardized PU–INU film was soaked in the solution and left at 37 °C on the incubator and in order to determinate the degradation rate (*D_r_*) at determinate elapsed times, it was represented in terms of weight loss (difference between initial and final weight of the samples) and calculated using a first order decay exponential differential Equation (2):

*D_r_* = *w_s_*/*w*_0_ = *e*^−*k*·t^, (2)

where *w*_0_ and *w_s_* are the initial weight and the weight at determinate time *t* and *k* a kinetic rate constant.

For the last two physical evaluations (swelling and degradation assessments), the samples were placed on sealed glass vials in order to avoid evaporation. Then, water excess on polymer surface was dried using absorbent paper and data is presented as a mean value of triplicate experiments.

### 2.4. Breast Cancer Application from PU–INU

#### 2.4.1. Drug Delivery System of DOXO

In order to determine the ability of PU–INU based materials as cancer drug delivery system, a model drug which has been studied and estimated through UV-Vis spectroscopy for this application, such as DOXO was used [23]. For all the test a standardized 0.5 cm^2^ film fraction of PU–INU and deionized water were used.

First, a known concentration of the drug within linear range of Lambert-Beer law (concentration range from 0.2 to 2.0 mg·mL^−1^) is measure at 482 nm using a 16,000-PC spectrophotometer (VWR, Wayne, PA, USA) with deionized water as blank and a calibration curve is recorded. DOXO test solutions were prepared from further dilutions of the initial 2.0 mg·mL^−1^ stock solution.

Then an initial DOXO solution of 2.0 mg·mL^−1^ was loaded into de PU–INU based materials through swelling until their maximum swelling ratio is reached, un-absorbed DOXO is calculated by measuring the absorbance relative to the prior calibration curve recorder under the same conditions and DOXO loaded was calculated by the difference of initial solution and unabsorbed DOXO. The described procedure allows to calculate the amount of drug loaded per unit of mass of PU–INU (*q*, mg·g^−1^) by using Equation (3):

*q* (mg·g^−1^) = (*C*_0_ − *C_e_*) * *V*/*w*, (3)

where *C*_0_ and *C_e_* are the initial and equilibrium DOXO concentrations (mg·mL^−1^), respectively, *V* the volume of the solution (mL), and *w* the mass of polyurethane used (g).

For drug release, PU–INU samples were placed in a glass vial containing the same volume of deionized water used for drug loading and after predetermined time intervals (approximately 20 min) the polyurethane were separated from stock solution and the percentage of released DOXO relative to loaded DOXO (*D_r_*, %) is estimated by comparing the absorbance measurement in UV–Vis with the plotted calibration curve and Equation (4):

*D_r_* (%) = [(*C_e_* − *C_r_*)/*C_e_*] × 100, (4)

where *C_e_* and *C_r_* are the concentration (mg·mL^−1^) of DOXO at equilibrium after drug loading of polyurethane and at a released time interval, respectively.

In order to determine the mechanism of drug release, the data obtain from release experiments were fitted according to the most relevant kinetics models [24], which are described hereby.

Zero-order model: A model used generally for drug dissolutions from pharmaceutical dosage that typically do not disaggregate and release the drug slowly. Therefore, the area of release is assumed as constant and no equilibrium conditions are reached. The model is simplified by Equation (5).

*Q_t_* = *Q*_0_ + (*K*_0_ · *t*) (5)

First-order model: The presented model for drug dissolution studies is the one used by Gibaldi and Perrier in which the adsorption and/or elimination of certain drugs is described. The theoretical concept of the model is difficult to describe but is usually expressed by Equation (6).

log *C* = log *C*_0_ − (*K*_1_/2.303) (6)

Higuchi model: This model was developed by Higuchi in 1963 in order to study the release of water-soluble and low-solubility drugs incorporated in solid/semisolid matrices. The simplified model is shown in Equation (7).

*f_t_* = *K_H_* · *t*^1/2^ (7)

Korsmeyer-Peppas model: A derived simple model by Korsmeyer in order to describe the drug release in a polymeric matrix, the *n* value was used by Peppas in order to characterize different release mechanism, this is summarized in Equation (8):

*M_t_* = *M*_∞_ · *a* · *t^n^* (8)

#### 2.4.2. Marker Clip for Biopsy

Additionally to PU–INU based materials synthesis, Fe_3_O_4_ iron oxide nanoparticles (IONP) were synthesized by co-precipitation of FeCl_3_ and FeCl_2_ iron salts (molar ratio 1:1), with 25% of citrate ions and NH_4_OH for maintaining pH synthesis at 9, as reported elsewhere [25] in order to form a radiopaque composite for evaluation. Briefly, 1% (*w*/*w*) of iron oxide nanoparticles respect PU–INU50 was mixed using mechanical stirring with low isopropanol content in order to form a paste. The paste was placed into a cast and isolated in a vacuum drying oven ADP 200C (Yamato Scientific Company LTD., Tokyo, Japan) at 40 °C with controlled atmosphere to evaporate the solvent and it solidified after 72 h. Finally, the composite was shaped into a non-biological form for discrimination (triangle with rounded edges).

First, iron oxide nanoparticles distribution on the polyurethane marker was measured using electron dispersive spectroscopy (EDS) in a Bruker XFlash 6/60 Silicon Drift Detector coupled to a Hitachi SU8230 cold-field emission microscope. Samples were placed on metallic stubs using a single gold layer conductor coating and all images were taken using secondary-electron detector at 30 keV at 15 mm work distance.

Then, radiopaque properties evaluation of the composite material was performed by implantation on a mammary gland model consisting on muscle and adipose tissue. Images were acquired using a dental imaging X-Ray RVG 5200 (Carestream Health Inc., Rochester, NY, USA) and, in order to suggest its use in stereotactic breast biopsy, a custom MATLAB script was made to observe its visibility in a 8-bit grayscale analysis as radiopaque scale, where the lowest value (black = 0) was considered as radiolucent and the highest value (white = 255) was considered radiopaque.

## 3. Results

### 3.1. Structural Characterization of PU–INU

**Figure 1:**
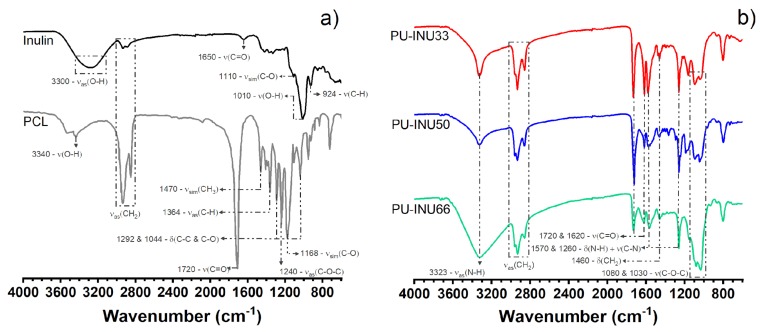
Fourier transform infrared spectroscopy (FT-IR) spectra of (**a**) INU and polycaprolactone diol (PCL-diol) as polyol precursors and (**b**) PU–INU base materials and their adsorption characteristic bands. Nomenclature corresponds to: ν = stretching, δ = bending, and ω = wagging.

Conversion of precursors into polyurethane and isocyanate group in PU–INU samples was monitored through FTIR spectroscopy in ATR mode since it has been suggested as useful tool for monitoring PU formation [26], as shown in Figure 1. The characteristic absorption bands from inulin were found from O–H polymeric asymmetric stretching at 3300 cm^−1^, C–H symmetric and asymmetric vibration from CH_2_ at 2934 and 2885 cm^−1^, C=O stretching was assigned to carbonyl band due acetylation or non-cyclic carbohydrates at 1625 cm^−1^, C–O corresponds to symmetric stretching from polysaccharide cyclic structure at 1110 cm^−1^, O–H bending vibration corresponds to carbonyl band at 1010 cm^−1^ and C–H twisting vibration corresponds to CH_2_ at 924 cm^−1^. From PCL-diol the bands at 3340 cm^−1^ from OH end group, bands at 2933 and 2857 cm^−1^ assigned to symmetric and asymmetric CH_2_ stretching, at 1720 cm^−1^ from carbonyl group (C=O), symmetric and asymmetric bending from CH_3_ and CH from 1470 and 1364 cm^−1^ respectively, from crystalline and amorphous region of PLC-diol related to C-C and C-O stretching at 1292 and 1044 cm^−1^ and bands at 1240 cm^−1^ from C–O–C asymmetric stretching and at 1168 cm^−1^ from C–O symmetric stretching are observed. Then, general examination of characteristic absorption bands of PU–INU based materials; N–H corresponds to asymmetric stretching associated to a primary amide at 3323 cm^−1^, C–H was assigned to symmetric and asymmetric stretching bond with the carbonyl at 2960 and 2858 cm^−1^, C=O was assigned to a stretching vibration of carbonyl of urethanes amide I at 1731 and 1680 cm^−1^, a combined N–H deformation and C–N stretching vibration corresponds to amide II and amide IV band at 1570 and 1260 cm^−1^, C–H deformation vibration from fructose ring is at 1460 cm^−1^ and C–O–C symmetric stretching vibration was found at 1080 and 1030 cm^−1^. It is important to notice that polyurethane synthesis was achieved and that there are no free isocyanate groups present in the samples because the adsorption band at 2250 cm^−1^ that corresponds to N=C=O from HDI does not appear in samples spectra.

**Figure 2:**
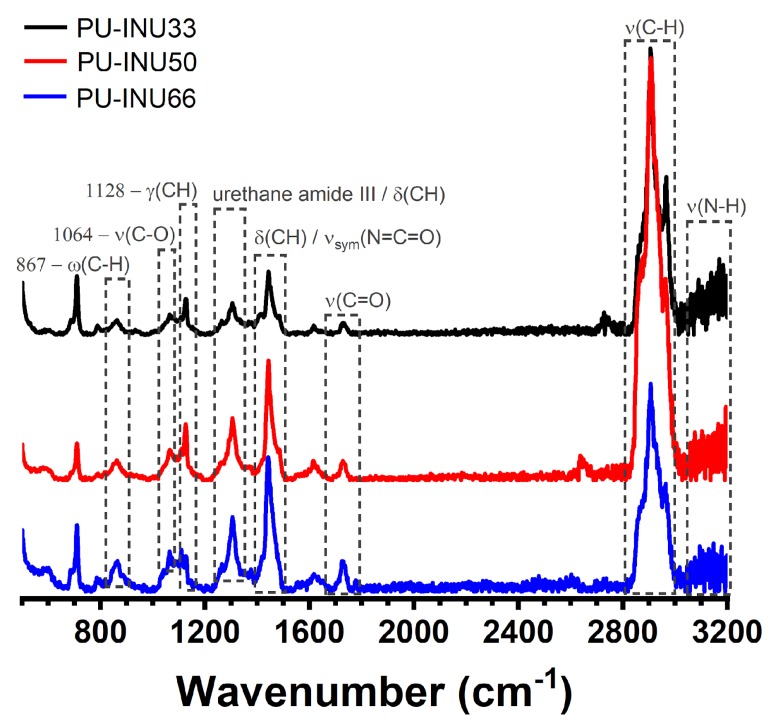
Raman spectra from PU–INU based materials and their adsorption characteristic bands.

As a complementary and supportive source of information for deeper qualitative insight of the vibrational modes and hereby the structure of the newly synthesized PU–INU polymer, Raman spectroscopy was performed as seen in Figure 2. It is important to highlight that only few studies deal with Raman spectroscopy characterization in addition to FT-IR and vice-versa for polyurethanes. Also, this technique is non-sensitive to humidity traces and based on the location, –OH groups of primary alcohols and secondary alcohols can be clearly differentiated [27].

The most important observed vibrational modes obtain from inulin are obtained at ~817 cm^−1^ from β-(1→4) glycosidic linkage from inulin, at 1059 and 1270 cm^−1^ from C–O–C, C–O, and C–C stretching and at 1333 and 1453 cm^−1^ from OH bending of a primary alcohol [27,28,29,30]. From PCL-diol, it can be divided into amorphous and crystalline section, in general are observed bands at 845/915 cm^−1^ V from C–COO stretching, 1098/1065 cm^−1^ (amorphous/crystalline) from C–O–C stretching, at 1287/1306 cm^−1^ corresponding to CH_2_ wagging vibrations (crystalline and amorphous), 1736/1725 cm^−1^ from carbonyl (C=O) stretching (amorphous/crystalline) and at 2920 cm^−1^ from C–H/CH_2_ asymmetric stretching (amorphous/crystalline) [31,32].

From a general survey of Raman scattering, obtained from our PU–INUs, are found several regions, the first at 3000~3200 cm^−1^ from N–H vibration (H bonded), from 2800~2910 cm^−1^ region assigned to symmetric and asymmetric C–H vibration, at 1725~1730 cm^−1^, a C=O vibration from ester groups and/or urethane from amide I was found; 1440~1445 cm^−1^ corresponds to a N=C=O asymmetric vibration and/or C–H bending vibration; 1303~1308 cm^−1^ was assigned to a urethane stretching and/or bending C–H vibration from amide III and 1125–1128, 1062–1064, and 863–867 cm^−1^ corresponds to C–H twisting vibration, C–O/C–O–C/C–C stretching vibration and C–H wagging vibration of ester groups and/or β-(1→4) glycosidic linkages, respectively. In addition, it is important to notice there is no sign of isocyanate asymmetric stretch from HDI at 2275 cm^−1^, indicating unformed isocyanate groups are not present in our PU–INU.

**Figure 3:**
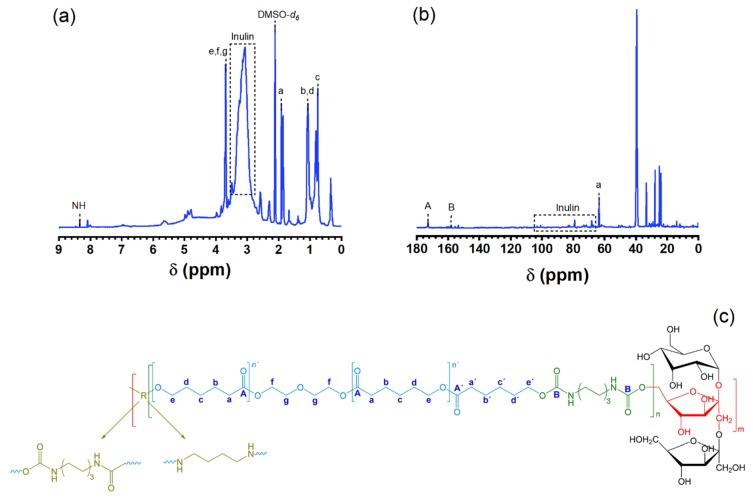
(**a**) ^1^H and (**b**) ^13^C spectrum of INU33 in dimethyl sulfoxide (DMSO)-d_6_ at 500 and 125 MHz, respectively. (**c**) Schematic representation of the suggested structure for PU–INU50 polyurethane. In red color are marked fructans from inulin where m ≈ 36, hexamethylene diisocyanate (HDI) moiety and the urethane linkage are marked in moss-green color, PCL-diol contributions are marked in navy-blue color, HDI or putrescine chain extensor of PU–INU50 are marked in olive-green color and in blue color are marked the letter that corresponds to the chemical moieties observed in NMR.

Since FTIR and Raman spectroscopy only provides qualitative results from the expected vibrational modes in polyurethane, rather than a quantitative and clear reaction mechanism, ^1^H and ^13^C NMR was performed to elucidate the structure (Figure 3).

From ^1^H spectrum (Figure 3a) is seen the presence of inulin and PCL-diol in the polyurethane structure, the peaks in the region between *δ* 4.82 and *δ* 2.63 ppm correspond to inulin structure and from PCL-diol the peaks at *δ* 4.02, 2.32, 1.53, and 1.26 ppm to different sp^3^ secondary carbons of its structure. Also, the hydrogen from amine and urethane group is observed at *δ* 8.29 ppm and the peak at *δ* 2.5 ppm correspond to solvent (DMSO-*d6*). Complementing, ^13^C spectrum (Figure 3b) exhibits peaks at *δ* 172.8 and 158.0 ppm corresponding to carbonyl groups of ester from PCL-diol and urethane from the polyurethane linkage respectively; the peak at *δ* 63.5 ppm was assigned to secondary carbons bonded to oxygen sp^3^ of the ester group (–CO–O–CH_2_–). Similar results are obtained from the ^1^H and ^13^C spectrums pf PU–INU33 and PU–INU66 and have consistent with the ones reported in literature [33,34,35]

### 3.2. Thermal Analysis of PU–INU

**Figure 4:**
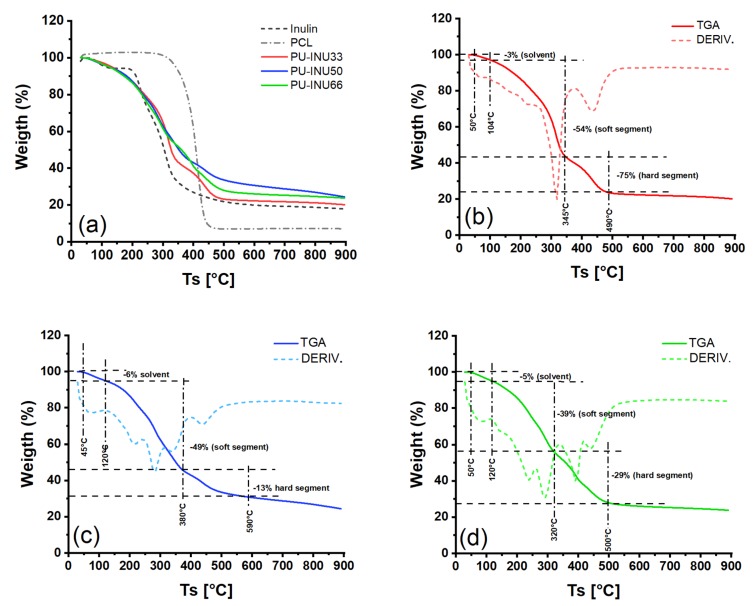
(**a**) Thermogravimetric analysis of polyol precursors (inulin and PCL) and synthesized PU–INU. Thermogravimetric analysis and its derivative (DTG) of (**b**) PU–INU33, (**c**) PU–INU50, and (**d**) PU–INU66.

The thermal behavior of the polyol precursors and the PU–INU can be observed in Figure 4. A weight loss of 10% was chosen in order to determinate the initial thermal stability of the components.

Figure 4a shows the thermogravimetric analysis of the polyol precursors, INU and PCL-diol. In the thermogram of INU, three characteristic thermal transitions are observed, the first from 25 to 180 °C related to moisture content of the sample, the second range from 180 to 240 °C corresponding to the initial inulin decomposition of the glycosidic linkages and followed to the last transition from 240 to 900 °C that goes from a continuous mass loss due to inulin decomposition and combustion [36,37]; and from the second (PCL-diol) it is seen only a single step thermal degradation corresponding to the backbiting of ester in a temperature range of 280 to 410 °C followed by the continuous decomposition and combustion of the sample [38].

For the PU–INU, additional to the graphs from Figure 4b to Figure 4d, detailed data of *T*_max1_, (maximum rate of degradation temperature in the first step), *T*_max2_, (maximum rate of degradation temperature in the second step), *T*_max-new_, (maximum rate of degradation temperature in the new step), and *W*_R_ (residue at 850 °C), are summarized in Table 2.

From data, it is observed that the solvent loss and free water by evaporation occurred at 80 and 150 °C with a weight loss of 3%, 6%, and 5% to PU–INU33, PU–INU50, and PU–INU66, respectively.

Between 200 and 250 °C, an acceleration of weight loss is produce, which is attributed to non-free water, trapped by weak bonds between the –OH groups of inulin/PCL and the polar domains of trapped water molecules.

At temperatures above 250 °C, it was observed the thermal degradation of the materials. As is known, polyurethanes have at least two-decomposition stages in their thermal degradation, where the first decomposition fraction (usually over 250 °C) in the polyurethanes chains is related to soft segments, which involve the thermolysis of urethane linkages, while the second decomposition fraction (usually over 350 °C) is related to the decomposition of macrodiol components and above 420 °C a gradual weight loss from continuous combustion of the polyurethane [20,21,26,39,40,41,42,43,44].

### 3.3. Physical Characterization of PU–INU

#### 3.3.1. Mechanical Performance of PU–INU

**Figure 5:**
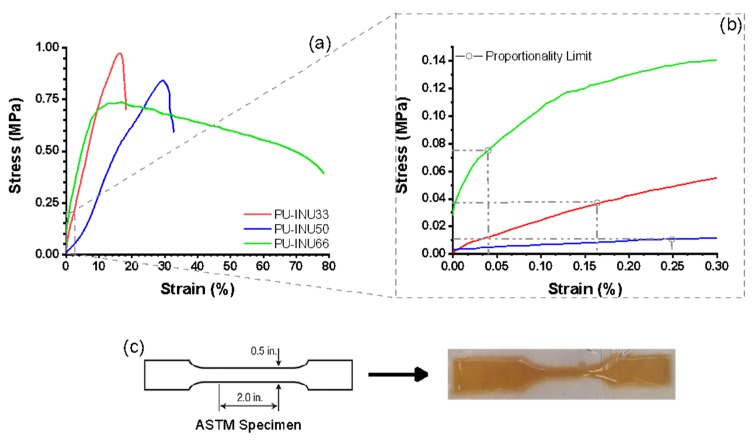
Mechanical behavior of the synthesized PU–INU. (**a**) Mechanical properties until maximum tensile strength was reached, (**b**) mechanical properties in the elastic region, and (**c**) example of the required specimen according to ASTM-D638-03 standard and the actual film.

Similarly, to thermal characterization, mechanical properties were studied as complementary information. Mechanical integrity of the material is necessary to withstand manipulation for the in situ applications, therefore, mechanical performance of synthesized PU–INU is performed and analysis of strain/stress data can be observed in Table 3 and Figure 5. From the strain/stress curves, values for elastic modulus (Young modulus) were calculated using a linear fit where the stress is directly proportional to the strain having the known behavior of proportionality limit (Figure 5b). In order to determinate the proportionally limit of the polyurethanes, the secant method was employed and the beginning and end of elastic modulus determination was from 0.05% to 0.1%, respectively.

As seen in Table 3, as inulin content is increased, the tensile strength value is reduced and strain percentage is increased. For the rest of the properties (yield stress, young modulus, and both proportionally limits) the values are also increased from PU–INU33 to PU–INU50 but the values are decreased from PU–INU50 to PU–INU66.

Considering the mechanical properties of the sample, they either can be used as drug delivery or marker clip, then sample PU–INU50 was selected to be tested as marker clip because it has the higher elastic modulus among all of the samples.

#### 3.3.2. Swelling Properties of PU–INU

**Figure 6:**
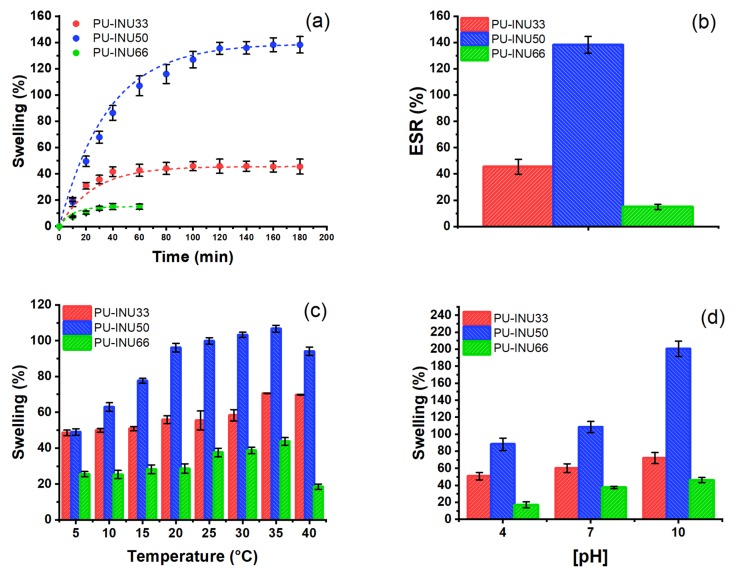
Swelling properties of the PU–INU’s: (**a**) swelling profile against time, (**b**) maximum swelling ratio against mol % of inulin per mol of polyol, (**c**) swelling ratio at different temperatures, and (**d**) swelling ratio at different pH.

Figure 6 shows the swelling properties of the synthesized PU–INU. The swelling behavior as a function of time at room temperature (20 °C) for the three samples is shown in Figure 6a and *ES_r_* is reached approximately at three hours after samples was left for water uptake. In Figure 6b, *ES_r_* is compared between samples and the one with the highest swelling capacity was PU–INU50, followed by PU–INU33 and PU–INU66 with an increase of 1.38-, 0.45-, and 0.14-times their own weight, respectively.

The hydrophobic/hydrophilic character of polyurethanes depends on its chemical structure and especially on the nature of physical and chemical interactions between urethane moieties from polar hard segments and polyol soft segments, where hydrogen bonds are predominant [45,46,47,48,49]. Therefore, it was expected that PU–INUs will exhibit hydrophilic properties, like when a hydrophilic chain extender is incorporated into the synthesis. In this sense, the behavior of samples PU–INU33 and PU–INU50 is reasonably explained by the increase in polar groups from the inulin polysaccharide structure. Nevertheless the swelling behavior of sample PU–INU66, presented a more hydrophobic character, instead of a highly hydrophilic polyurethane which cannot be explained by a greater presence of polar groups.

Figure 6c shows swelling behavior as a function of temperature at equilibrium swelling ratio for each one sample and as can be seen, the samples show a classic swelling behavior dominated by the solubility of the polar groups, thus, for higher temperatures, a tendency for greater swelling is observed.

For swelling, as a function of different pH conditions at equilibrium, swelling ratios are different, as observed in Figure 6d and that clearly there is a trend that indicates greater swelling at basic pH.

#### 3.3.3. Degradability Assessment of PU–INU

**Figure 7:**
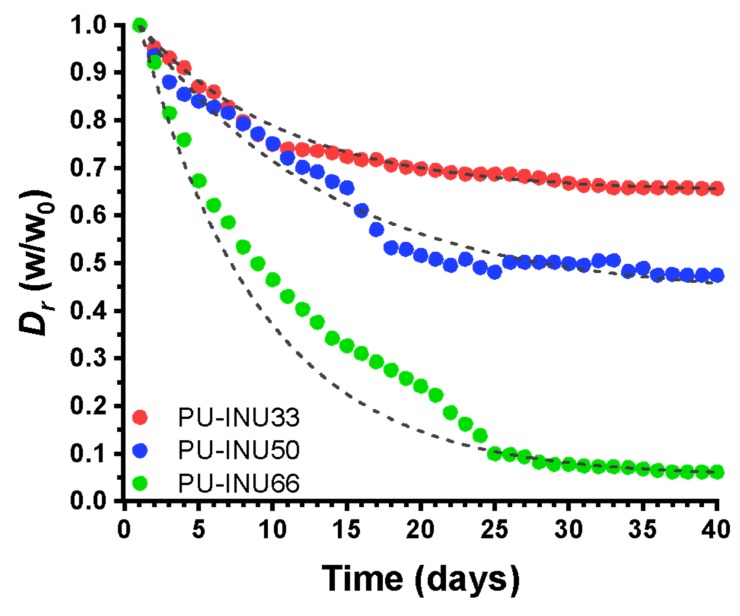
Degradability rate under specific pH and temperature conditions (7.4 at 37 °C) of the different PU–INU synthesized.

The results for the PU–INU degradation under specific pH and temperature conditions could be observed in Figure 7 and data is adjusted using the model presented in Equation (6) and Table 4 summarizes the degradation rate and the final weight loss percentage after the elapsed time of each one of the samples.

### 3.4. Breast Cancer Application of PU–INU

#### 3.4.1. Drug Delivery of DOXO

**Figure 8:**
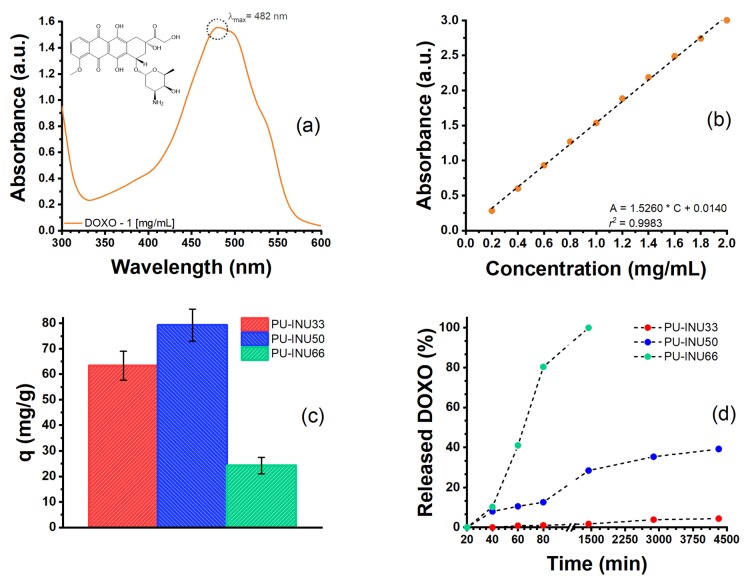
Application of PU–INU’s as drug delivery system of doxorubicin hydrochloride (DOXO). (**a**) DOXO UV–Vis spectra at 1 mg*·*mL^−1^, (**b**) DOXO calibration curve with a linear fit of *R*^2^ > 0.99, (**c**) sorption capacity (q, mg*·*g^−1^) of synthesized PU–INU, and (**d**) drug released relative to drug loading concentration of PU–INU.

Complete analysis of the inulin-based polyurethanes as a drug delivery system can be observed in Figure 8. DOXO UV-Vis spectra (Figure 8a) shows the maximum absorbance was taken at 482 nm in order to construct a calibration using different concentrations (Figure 8b) of the drug, which presented high correlation values.

For DOXO loading into the PU–INU matrix, results are in accordance with swelling since PU–INU50 is the sample which has the higher sorption capacity of the drug (79.25 mg*·*g^−1^), followed by PU–INU33 (63.74 mg*·*g^−1^), and PU–INU66 the one with the lowest (24.27 mg*·*g^−1^). For DOXO delivery it is observed that PU–INU66 is the sample with the fastest release within an interval of five hours to deliver all the loaded drug against the amount of time required for samples PU–INU33 and PU–INU50 to release its 4.39% and 39.22%.

In order to describe the physico-chemical phenomena of how the drug delivery rate is given, the use of four different theoretical models was needed and the results obtained are summarized in Table 5.

#### 3.4.2. PU–INU50 Application as Marker Clip

**Figure 9:**
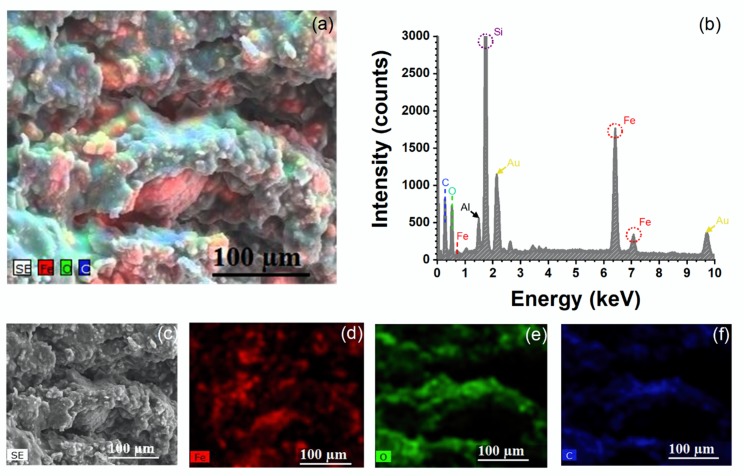
Elemental mapping of the Fe_3_O_4_ iron oxide nanoparticles (IONPs) on PU–INU50. (**a**) Element (Fe + O + C) overlay distribution on the polyurethane, (**b**) complete electron dispersive spectroscopy (EDS) spectra of all elements distribution, (**c**) scanning electron microscopy (SEM) image and (**d**–**f**) element mapping of Fe (red), O (green), and C (blue).

An elemental mapping of the marker clip prototype was analyzed using SEM images and energy dispersive spectroscopy (SEM-EDS) in order to observe the synthesized IONP’s distribution on PU–INU50. Figure 9a shows the complete elemental distribution and Figure 9b shows its corresponding EDS spectrum, which confirms the existence of iron (Fe), oxygen (O), and carbon (C) signals in the polyurethane marker prototype. The rest of the signals correspond to Al from stub, Si from silicon plate support, and Au from a deposited layer of sample preparation. Figure 9c shows the SEM image of the polyurethane marker surface morphology and Figure 9d–f shows the individual EDS mapping of element distributions of Fe (red), O (green), and C (blue), respectively, of which it can be inferred that IONP’s are well distributed on the PU–INU50 surface.

**Figure 10:**
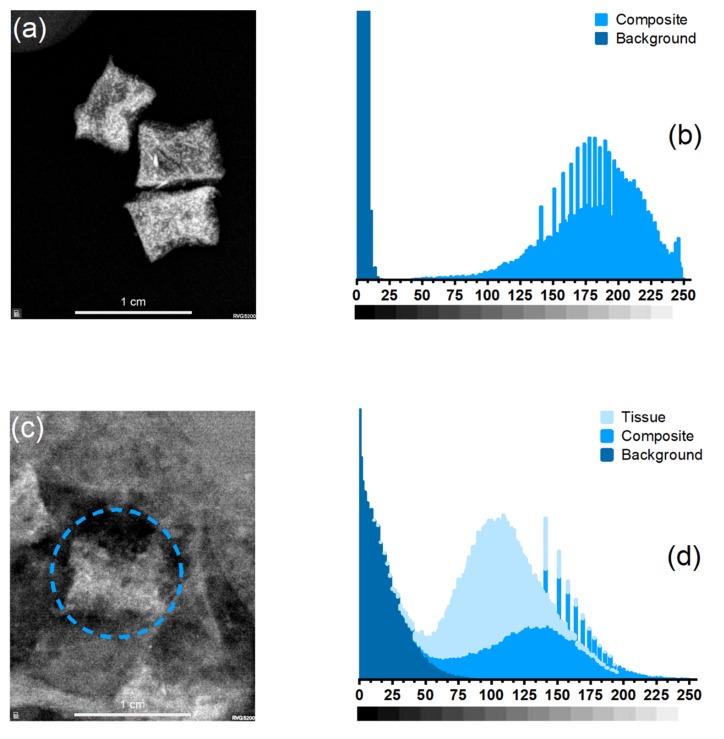
Images of radiopaque validation of PU–INU50. (**a**) X-Ray imaging and (**b**) grayscale histogram of the prototype marker, (**c**) X-Ray imaging, and (**d**) histogram of the prototype marker inside model tissue.

On the other hand, verification for radiopaque properties was made (Figure 10) and statistic values of the grayscale analysis are presented in Table 6. In Figure 10a, it is notable that the composite shows a regular distribution of the nanoparticles as seen in the EDS elemental distribution of Fe (Figure 9d) and from the gray scale histogram and data (Figure 10b). The composite can be distinguished using X-Ray imaging, an essential feature to material potential use in imaging for breast pathologies in general. Then, a test in a model tissue was performed (Figure 10c) and there is no obvious distinction between the material, tissue and background but it can be located in the image observing it carefully. This is clear by analyzing it using grayscale (Figure 10d), the device could not be easily identified because of its histogram, the tissue and the background are overlapped, but from the data, the average value of the composite is slightly higher than the tissue and that is why it is possible for marker identification which is marked in Figure 10c inside a blue circle.

## 4. Discussion

The synthesis of inulin-based polyurethanes (PU–INU) proceed successfully since in the spectroscopy characterization the vibrational modes related to the urethane groups are always present (C=O and N–H). From FT-IR spectra, there is a match between all bonds and groups of polyol precursor mainly to the –OH and CH_2_, C=O, C–C, C–O, and C–O–C vibrations, with slights difference in wavenumber since inulin is a cyclic polysaccharide and PCL-diol an aliphatic polyester with a linear structure, as an example the C–O–C vibration is presented at 1160 and 920 cm^−1^ for PCL-diol and inulin, respectively. Also, an –OH change in bandwidth is presented indicating reaction with –NCO groups, with a decreased also in the carbonyl group (C=O, ~1620 cm^−1^) related to formation of urethane. Additionally, to the typical vibration of the precursor shift in the wavelength of the PU–INU can be attributed to structural changes of polyols precursors due formation of new bonds, crosslinking, or new intermolecular interactions.

Raman characterization serves as a complement to the observed vibrational modes discussed in FTIR with the difference that the second could give us insight of environmental changes and through the use of the former it is clearer how the backbone of the PU–INU changes as INU content is increased and PCL-diol content is reduced. It is primarily observed that the N–H stretching region is reduced, the second peak of C–H stretching located at 2966 cm^−1^ is less obvious and is shifted to 2961 cm^−1^, there is an increase intensity in the C=O vibration located at 1730 cm^−1^, at 1445 cm^−1^ a shift is presented from the C–H stretching from primary alcohol of inulin (1453 cm^−1^) and also from the same vibration presented in PCL-diol (1440 cm^−1^) which intensity is increased since it has more hydroxyl terminations in comparison and can be related with the increase presented at 1304 cm^−1^ related to urethane vibrations, shifted from the 1333 cm^−1^ bending of the primary alcohol of inulin since it has more moieties available to react and also the doublet from the wagging of CH_2_ from PCL is being diminished. The vibration at 1128 cm^−1^ is also reduced from the twisting vibration of the ester groups present in PCL-diol, the peak at 1064 cm^−1^ is shifted from inulin and PCL carbonyl vibration (C–O–C, at 1059 and 1065 cm^−1^, respectively) and also is increased due the increase content of aldehyde groups of the fructose chains of inulin and finally the vibration of 863 cm^−1^ is shifted from the band of β-(1→4) of inulin and the band of 863 cm^−1^ from α-carbon of ester group of PCL-diol with an increment in it related to the described glycosidic linkage.

Finally, NMR gives insight that PCL-diol and inulin reacted with the diisocyanate forming the urethane group, but it does not give us the structural conformation, so is suggested that inulin and PCL-diol in the polyurethane structure are in a randomized arrangement. It is important to highlight that since there is an isocyanate groups excess in the reaction, it can react with majority of the hydroxyl groups present in inulin as proposed in Figure 3.

There is no appearance of isocyanate groups from the precursor in the performed spectroscopies, which is of great importance for the required application since this chemical moiety are of high toxicity [39] and also it serves as an indicator that the cleansing process of unreacted chemical species of the PU–INU proceed correctly and residues were completely washed.

For the desire applications the PU–INU are thermally stable (around 37 °C) as observed in Figure 4. Thermal gravimetric analysis was used as a complementary technique to verify the reaction between the polyol precursors and the isocyanate from a physical property point.

It is observed that as INU content in PU–INU is increased, the moisture content is increased but a slight difference in PU–INU50 and PU–INU66 is observed (6% against 5% in weight), giving an insight that there is more –OH moieties available for hydrogen bonding within the samples. It is also observed that for higher content there is a change in the number of thermal transitions from three to four. The first step is related to moisture content, the next from the first decomposition process of the polyurethane (soft segment), an intermediate that appears from non-reacted or hydrogen bonded inulin and the last from the second decomposition step of the polyurethane (hard segment). It is observed that there is deviation from the polyol precursors and from each of the samples in where the temperature of the process is centered indicating and reinforcing that the structure of the PU–INU backbone is changed.

Specifically, for PU–INU33 the first step decomposition transition of the polyurethane backbone was at 319 °C, the second step decomposition was 425.4 °C with *W_R_* of 20.23%, which are results consistent with data reported previously [44,50,51,52,53]. Therefore, an inclusion of 33 mol% inulin did not generate significant changes in the behavior of a polyurethane.

Then, for PU–INU50 and PU–INU66, its first step decomposition was at 276.9 and at 282.7 °C, respectively, while its second step decomposition was at 425.8 and 425.5 °C, respectively, i.e., that lower thermal stability in its soft segments was presented, while the stability of the hard segments was preserved. Those findings were expected because it is known that the inter-urethane hydrogen bonding plays a significant role in the thermal stability of segmented PU, and when introducing inulin chains in the soft section of the polyurethane, thermal decomposition was expected at temperatures close to the degradation of the inulin fructose chains [53,54,55,56].

For PU–INU50 and PU–INU66 the intermediate degradation step was presented at 328.2 and at 390.2 °C, respectively, which is related to the thermal decomposition of non-bonded or hydrogen bonded inulin fructan chains.

In order to complement the analysis of polyol precursors on the final physical properties of the PU–INU, mechanical performance was performed. In this regard, the mechanical performance of the PU–INU´s was comparable with human skin elastic modulus (129 KPa) [57]. Stiffening was similar to human skin when breast cancer is presented (3 KPa on ultimate tensile strength and 45 KPa for elastic modulus) [58,59], in consequence the materials can withstand application.

Observing data in Table 3, it is noticeable how tensile strength and elongation are increased with inulin content as shown from PU–INU50 to PU–INU66. The results suggest that as the content of inulin is increased, it serves as a high hydrogen-bonding agent, changing the mechanical behavior of the material.

Crosslinking due to the non-bonded inulin not only affects the mechanical behavior but the swelling properties are also modified with a greater degree of cross-linking because it increases the hydrophobicity of polyurethanes materials [47,60,61]. Accordingly, the swelling behavior showed (Section 3.3.2, Figure 6a) in samples PU–INU33 and PU–INU50 can be explained by the increase in polar groups from the inulin polysaccharide structure, as already mentioned. However, when the concentration of inulin in the reaction is close to 66% (sample PU–INU66, Figure 6b), it is obtained a hydrophobic polyurethane due to the cross-linking caused by a higher concentration of non-bonded inulin (free inulin), within the structure of the polyurethane material PU–INU66. For the samples with relatively low inulin content (PU–INU33 and PU–INU50) it is observed that both have a similar mechanic and thermic properties compared to a hydrophilic polyurethane synthesized with PCL-diol or even as a polyurethane-based on diisocyanate with ether group such as poly(oxyethylene) glycol [49], this due to the presence of inulin chains. These polyurethane materials can uptake water by hydrogen bonding since the molecule has available link sites in contrast with the sample with the highest inulin content (PU–INU66) which has less amount of available link sites due to the presence of more free inulin that could also hydrolyze and make the structure unstable [60,61,62,63,64]. PU–INU50 presented a balance on mechanical and thermal properties which is reflected in a balanced structure with inulin in urethane linkage and non-bonded which gives the highest swelling capacity of all the samples.

From swelling at different dependent conditions it is seen that for temperature, there is a maximum swelling is at 35 °C and from it to 40 °C the samples suffer a loss in swelling capacity which can be used for drug delivery since it occurs in an interval which human temperature is located (37 °C). For pH-dependent behavior, it is observed that in acid pH for all the samples the swelling degree is the lowest, due to the inulin hydrolyzation, the free sugar concentration increases (above 3%) and it produces that the inulin swelling capacity decreases [64,65]. For neutral and alkaline, pH swelling degree increases due to the enhancement in intermolecular interactions. According to previous knowledge, the inulin structure is stable in neutral and alkaline pH, therefore, reducing-sugars concentration decreases below 3%, improving swelling capacity [65].

Regarding degradation assessments, it is observed that as inulin content is increased, weight loss is increased because the polyurethane structure is more exposed to hydrolysis since it has more hydroxyl groups present from inulin structure. This is observed in Table 4, which summarized the analysis after two months under the specified conditions (pH 7.4 and 37 °C) and the sample with the highest percentage of weight loss was PU–INU66 (59.12% of its total weight), which also has the highest constant rate of degradation (*D_k_*). 

Considering DOXO structure in drug release, there is a possibility of intermolecular linkage with the polyurethanes, so release mechanism could be dependent on the cleavage of hydroxyl and urethane groups to release DOXO molecules and the second the diffusion of DOXO molecules from the polymer matrix to the surrounding medium. The use of the mathematical model help to predict the release of this particular systems, from the performed studies is observed that not all the samples fit to the models proposed for drug, specially sample PU–INU66 which has in all cases correlation values were *R*^2^ < 0.5 indicating that other theory like Hopfenberg model for heterogeneous surface eroding devices could be of great use since from this sample is observed the greatest material degradation giving insight of this kind of released behavior. For the rest of sample is observed that more than one of the proposed models are well fitted (correlation values of *R*^2^ > 0.9) indicating that drug delivery for this system is through a complex process.

For sample PU–INU33, both zero-order kinetics and the Higuchi model have adequate and closed correlation values (*R*^2^ > 0.9), this is indicative that there are two simultaneous or complemented process involve, the first is a case II transport, in other word, drug concentration exceed drug solubility in the polyurethane indicating drug concentration is constant at the inner surface and release is replaced by partial dissolution of drug crystals, the second process is Fickian diffusion that follows or complement drug transport. This kind of release are is ideal for long-term pharmacological action. At last, for sample PU–INU50 the model with the highest correlation value (*R*^2^ > 0.98) was the Korsmeyer–Peppas model, the *n* value of 0.3192 is an indication that the transport of the drug is by Fickian diffusion [66], this is confirmed since the Higuchi model is also well fitted (*R*^2^ > 0.93).

Aside from the drug released model proposed there is a noticeable difference in drug release from sample PU–INU66 which can be due to inulin crosslinking density of the sample, in e.g., several reports have described how the high crystalline structure and hydrophobic behavior of PCL-diol make its degradation slower when compared with other aliphatic polyesters [67]. Because of this, it is always recommended to make copolymerization with monomers of greater hydrophilic or make crosslinked structures [68], the inulin crosslinking density plays and important role since crosslinking decreases PCL-diol crystallization and permits hydrolysis in amorphous sections enhancing degradation. The hydrolysis in amorphous sections facilitates drug release from PCL-diol based polymers [67,69,70,71].

Hence, crosslinking density from inulin and considering that the amount of free inulin is greater by INU-66 compared to INU-33 a combined effect is seen making all drug to be released in less time in samples with higher amount of inulin, playing an important role in the stark difference in the release kinetics and the total amount of the released drug between samples INU-66 and INU-33

For radiopaque test, as mentioned, PU–INU50 was selected for screening since it has the lowest mechanical deformation and the highest elastic modulus with a mid-term degradation in the simulated conditions. The performed radiopaque test supports the idea that the material can be shaped in other geometry, as a cylinder for example, and be tested in an animal specimen to verify its radiopacity, durability, and degradability over time. Also, the composite can be tuned with the inulin content, to be able to degrade in a determinate time and since the nanoparticles are well distributed the radiopacity would not be lost drastically and also as shown can release a chemotherapeutic drug if needed. Also, the content of iron oxide nanoparticles can be increased to improve radiopacity since the used concentration is still recommended as non-toxic [72], but still further assays need to be done to validate their released products upon degradation are non-toxic in an in vivo model as identifying their lysosomal localization.

## 5. Conclusions

In this work, it is reported that the synthesis of a novel polyurethane based in inulin/polycaprolactone diol and isocyanate as precursors was confirmed by FT-IR, Raman, NMR spectroscopy, TGA, and mechanical properties studies.

Regarding swelling, it is observed that hydrogen bonding of non-reacted inulin into the polyurethane at determinate concentration do not permit the swelling of the sample and swelling properties at different conditions is governed mostly by the inulin in the structure of the polyurethane but it is interesting how the un-swelling from 35 to 40 °C is important since this properties could be used for drug delivery and studies in between this interval are needed to find the exact temperature where this change is presented. It is important to highlight that all the samples are degradable in temperature and pH simulated physiological conditions.

As increasing inulin (decreasing PCL-diol) content of the PU–INU, the drug release mechanism is complete modified passing from dissolution of the drug inside the polymer, to diffusion to at last erosion and also the amount of released drug against time. This allow for these particular types of polyurethanes to be designed according to the amount, time, and type of drug to be given according to the zone or type of breast cancer presented in order elevate the effectiveness and patient well-being.

Radiopacity tests showed that the material has potential applications to be used as a biopsy biomarker, since it is also biodegradable. However, it is recommended to do more tests to establish that it can be used as a biopsy biomarker of the mammary gland.

The scientific findings shown in this report, could be the starting point for a technological development of greater impact on the fight against the breast cancer.

## Figures and Tables

**Table 1:** Molar ratios between polyol:isocyanate precursor and the amount of precursor used for synthesized inulin-based polyurethane (PU–INU).

Sample	OH:NCO	Per Mole of OH INU:PCL	HDI [mg]	INU [mg]	PCL-diol [mg]
INU33	1:3.4	1:2	183.969	457.88	437.67
INU50		1:1		915.57	218.83
INU66		2:1		686.81	328.26

**Table 2:** Temperatures related to the different degradation step process in the PU–INU based materials.

Sample	T_max1_ [°C]	T_max-new_ [°C]	T_max2_ [°C]	W_R_ [%]
PU–INU33	319.0	---	425.4	20.23
PU–INU50	276.9	328.2	425.8	24.52
PU–INU66	282.7	390.2	425.5	24.02

**Table 3:** Mechanical properties values obtained of the synthesized PU–INU.

Sample	E ^1^ [MPa]	YS ^2^ [MPa]	TS ^3^ [MPa]	SA ^4^ [%]	SA-PL ^5^ [mm/mm]	S-PL ^6^ [MPa]
PU–INU33	20.88	0.24	0.97	18.26	0.16	0.03
PU–INU50	50.31	0.28	0.84	32.81	0.25	0.01
PU–INU66	3.18	0.03	0.74	96.30	0.04	0.07

^1^ Young modulus, ^2^ Yield Stress, ^3^ Tensile Strength, ^4^ Strain, ^5^ Strain Proportionality Limit, ^6^ Stress Proportionally Limit.

**Table 4:** Results of degradation rate in physiological conditions (pH 7.4 and 37 °C) and weight loss percentage after an elapsed time of two months of experiments.

Sample	*D_k_* (24 h^−1^)	*R* ^2^	Weight Loss (%)
PU–INU33	0.1028	0.9795	34.43
PU–INU50	0.077	0.9671	52.47
PU–INU66	0.1216	0.9411	93.85

**Table 5:** Mathematical model correlates coefficients and release exponents for PU–INU.

Model	Parameter	Sample		
		PU–INU33	PU–INU50	PU–INU66
Zero Order	*R* ^2^	0.9198	0.8388	0.3524
	*K*_0_	0.0009	0.0081	0.0499
First Order	*R* ^2^	0.2741	0.7812	−0.0136
	*K*_0_	0.0003	1.0491	0.0003
Higuchi	*R* ^2^	0.9206	0.9353	0.4542
	*K*_0_	0.0665	0.5705	2.3797
Korsmeyer-Peppas	*R* ^2^	0.4476	0.9846	0.2067
	*K*_0_	0.0088	1.1073	5.2722
	*n*	0.7600	0.3192	0.4371

**Table 6:** Grayscale data from radiopaque analysis done to the composite X-Ray images.

	Minimum (bit)	Maximum (bit)	Average (bit)	Relative Radiopacity against Background (%)
Background Figure 9a	0	19	10	0
Composite Figure 9a	42	250	146	93.20
Background Figure 9c	0	123	62	0
Composite Figure 9c	31	249	140	55.72
Soft Tissue Figure 9c	11	195	103	39.81
